# Promoting adolescent mother self-efficacy for parenting roles, and self-care after childbirth: protocol for a mixed methods study

**DOI:** 10.1186/s12978-023-01679-9

**Published:** 2023-10-04

**Authors:** Samaneh Youseflu, Shahnaz Kohan, Firoozeh Mostafavi

**Affiliations:** 1 Department of Midwifery and Reproductive Health, Reproductive Sciences and Sexual Health Research Center, Isfahan University of Medical Sciences, Isfahan, Iran; 2 Department of Midwifery and Reproductive Health, Faculty of Nursing and Midwifery, Isfahan University of Medical Sciences, Isfahan, Iran; 3 Department of Health Education and Promotion, School of Health, Isfahan University of Medical Sciences, Isfahan, Iran

**Keywords:** Adolescent mother, Interventional program, Self-efficacy, Mixed method study, Iran

## Abstract

**Background:**

Pregnancy, and childbirth can encounter many challenges for the teen mother, family, and health system. The simultaneity of two transition periods, i.e. transition to adulthood and post-partum period may disrupt the acceptance of new roles. Lack of knowledge, information and life skills in managing this situation may threaten the physical and mental health of the mother, and child, as well as reduce the level of role adaptation. One way to increase women's empowerment in postpartum care is self-efficacy promotion training program. The current mixed methods study will be conducted to provide an interventional program sensitive to the culture of the Iranian society for adolescent mothers to improve their self-efficacy.

**Method:**

This study will be conducted as an exploratory sequential mixed methods study (Qual-quan) based on a pragmatism paradigm in four phases. In the first phase, a qualitative study will be performed using a directed content analysis method to explore the needs and strategies related to promote postpartum self-efficacy from the viewpoint of teen mothers, their family, healthcare providers, and policymakers. In the second phase, using a scoping review, self-efficacy promotion strategies, and postpartum care service packages, programs, guidelines, and protocols in other countries will be identified. In the third phase, with integrating the results of qualitative phase and scoping review, the first draft of program will be developed, and validated by an expert panel based on the Delphi approach in three rounds. In the last step, the effectiveness of the interventional program will be evaluated on postpartum self-efficacy of teen mother.

**Discussion:**

Developing an interventional program that includes teen mother’s experiences, evidence based practice principles, and health staff opinions in a distinct cultural and social context can supply new direction to lead manager, policymakers, and other health care provider to improve the maternal self-efficacy in infant, family, and self-care with considering their age characteristics.

## Introduction

Adolescence as a transition period is considered one of the most sensitive life stages for adolescents and their families [1]. Upon entering this period, they experience rapid physical, cognitive, psychological, and social growths that can influence their feelings, thinking, decision-making, and their interactions with the world around them [2]. Since the transition from childhood to adolescence is associated with many psycho-social pressures; any additional stress such as early marriage, pregnancy, childbirth, and adaptation to motherhood roles can double the burden of psycho-social pressure imposed on them and as a result interfere with their roles as a daughter, student, wives and mothers, as well as affect their relationships with peers, family, and society [4].

Investing in the health of adolescents is also important from a demographic rationale. Adolescents make up a fifth of the world’s population, and 85% of them live in developing countries [6]. It is estimated that about 12 million girls aged 15–19 years and at least 777,000 girls under 15 years give birth annually in developing countries; which in most cases (at least 10 million) is unplanned. Also, the average global birth rate per 1000 women aged 15–19 is 65, this number in Iran is 40.81 birth [7]. It is predicted that the trend of teen pregnancy in the world will increase and reach at least 1.15 million by 2030 [8].

Adolescents make up about a fifth of Iran’s population, and according to the report of the Iran Statistics Center, and Office of Women’s Studies and Research in 2016, the marriage of men aged 20–24 to women aged 15–19 accounted for the largest number of registered marriages. Also, only 33 percent of them used contraceptive methods and one-third of them got pregnant in the first year after marriage [9].

Pregnancy and childbirth in this age group are important issues that can threaten their physical and mental health, and increase the risk of mother and child death [10]. The World Health Organization (WHO) mentions teen pregnancy as the number one killer of adolescents; worldwide, one out of every 3900 teen girls dies from the consequences of pregnancy and childbirth, and in developing countries, this number is 250 [11].

In addition to the negative physical/psychological consequences of teen pregnancy, adolescents are particularly vulnerable to postpartum changes. Transition to the postpartum period and accepting a new baby, as well as adaption to parental roles, can be a stressful experience for all parents, regardless of age and childbearing background, and requires adaptation to the upcoming changes [13].

Teen mothers are more confused in accepting multiple roles, because in addition to the underlying factors of teen pregnancy (e.g., poverty and low socioeconomic level), the normative changes that occur during adolescence such as the formation of individual identity, tendency to authority, and also the inability to negotiate in one’s relationships with the family and others can be a big obstacle in adapting to the parental role [14].

Due to the importance of the postpartum period in mother and child health, many international guidelines (e.g., NICE, WHO, and ACOG guidelines) have been developed for better postpartum care [17–20]. The main approach of all existing guides is to provide services and health promotion and does not pay attention to improving the self-efficacy and empowerment of mothers in the postpartum cares. Moreover, the content of care for all mothers with different characteristics and in all age groups was similar to a large extent.

The results of various studies indicate that the postpartum period of teen mothers should be viewed differently. Various studies have proposed postpartum care in the form of youth-friendly services, school-based postpartum care, home visit, web-based care, and group care models, and the nature of all models is women empowerment [21–25]. From the perspective of the WHO, empowerment as the heart of health promotion is a process through which people gain more control over decisions and activities affecting their health. One way to increase women’s empowerment is self-efficacy promotion training program [26, 27].

Self-efficacy is considered a fundamental concept in maternal care because it emphasizes the importance of self-management and empowerment [28]. Bandura’s self-efficacy theory states that people generally take action if they believe that they can do something, and if they believe that they will fail in doing something, they avoid it [29]. Self-efficacy promotion requires four main resources, including successful experiences of the individual, social modeling, social persuasion, and psychological responses, which should be considered when designing interventions [30].

Adolescents, due to their age characteristics, need to receive their information and special care, most of which is not provided in the current health system designed for adults. Therefore, the provided services should be sensitive and consistent with their biological, psychosocial, and cognitive needs. Thus, the existence of a comprehensive program to consider all aspects of adolescents' characteristics, and their needs, under the socio-cultural, and health structure of Iranian society, seems to be essential. Therefore, the current study is designed to develop a program for improving the self-efficacy of adolescent mothers in child, and herself cares during the postpartum period, using a sequential exploratory mixed method study.

### Objectives

The objectives of the four stages of the study are as follows:

#### The first phase: qualitative study

1.1. Identifying the needs and strategies for self-efficacy promotion of teen mothers in child, and herself cares during the postpartum period, from the perspective of the teen mother, health care providers, managers, and policy-makers with a directed content analysis approach.

#### The second phase: scoping review

1. Determining postpartum care service packages, programs, guidelines, and protocols in the other countries.
2. Extracting postpartum self-efficacy promotion strategies and recommendations from the literature.

#### The third phase: designing a postpartum self-efficacy promotion program

1. Designing the initial self-efficacy promotion program for teen mother by integrating the results of qualitative phase and scoping review.
2. Validating the developed postpartum self-efficacy program for teen mothers by expert panel.

#### The fourth phase: implementation of the postpartum self-efficacy promotion program for teen mothers

1.1. Determining the effect of a comprehensive postpartum self-efficacy promotion program on the self-efficacy score of teen mothers**.**

## Materials and methods

The current study will be conducted as an exploratory sequential mixed methods study (Qual-quan) based on a pragmatism paradigm in four phases. In the first phase, a qualitative study will be performed and the conclusion resulting will be analyzed using a directed content analysis method to determine the needs and strategies related to promoting postpartum self-efficacy of adolescent mothers in infants and herself cares from the viewpoint of teen mothers, their family, healthcare providers, and policymakers. Data will be collected via semi-structured individual in-depth interviews and taking field notes. Participants who are eligible for participation will be selected in a purposive approach and with maximum variation. Sampling will be continued until data saturation. In the second phase, using a scoping review, self-efficacy promotion strategies, and postpartum care service packages, programs, guidelines, and protocols in other countries will be identified. In the third phase, the results of the qualitative phase, in addition to the scoping reviews, will be used to design an appropriate interventional program. Then, this proposed program will be validated by an expert panel including obstetricians, midwives, and reproductive health professionals based on the Delphi approach in two, or three rounds, and then according to expert opinions, a comprehensive postpartum self-efficacy program will be developed for teen mothers. In the last step, the effectiveness of the interventional program is evaluated on postpartum self-efficacy. The collected data will be entered into SPSS statistical software version 22 and will be analyzed by descriptive-analytical statistics.

### Phase I: qualitative study

At this phase, the researcher is trying to explore the needs, barriers and strategies for self-efficacy promotion of teen mothers in the postpartum period to design an interventional program for their participation. This study will be carried out using a directed content analysis method based on Bandura’s self-efficacy theory.

### Study participation

In the qualitative part of the current study, participants will include adolescence mother aged between 15 and 19 years with the maximum variation of the age, mode of delivery, gravidity, time elapsed since delivery, social class, economic status, residency, and educational level. Also, health service providers who have experience in providing health care and treatment services to teen mothers including gynecologists, reproductive health specialists, general practitioners, midwives, psychiatrists, psychologists, family health experts, social workers, as well as, maternal health policy makers, with at least two years of working experience in this field, would be recruited for the study with informed consent.

In order to access the teen mothers, after obtaining the necessary permits, they will be contacted using the Sib system, which records information on the mothers’ pregnancy, delivery, and postpartum care, as well as the mothers’ contact numbers and addresses. After explaining the objectives of the study, we ask them to participate in our study. Also, in the health care centers that had more teen mothers, their health care providers will also be interviewed.

### Research environment

The interviews will be performed in coordination with the participants' views at the time and place of the participants’ desire including hospital, health care centers, midwifery or gynecologists’ offices, deputy of health and medical centers, work places, home, university, etc.

### Sampling method

Participants will select through purposive sampling, and the interviews will conduct with those who had the inclusion criteria after receiving their informed consent. The interviews will be continued until data saturation, and it will stop if data saturation occurs.

### Inclusion criteria for teen mother

- Willingness to participate in the study, and providing the informed consent
- Age between 15 and 19 years
- The elapsed time from childbirth between 42 days to one year
- Having a healthy infant without any congenital disorders
- Iranian Nationality
- Not history of well-known psychiatric disorder requiring medication
- Being able to participate in the interview physically or mentally

### Inclusion criteria for health care provider

- Having at least 2 years of working experience in healthcare centers for healthcare providers, and 3 years for policymakers, which are all in the field of providing services to teen mothers.
- willingness to participate in the study with informed consent

### Data collection process

After approving the protocol of study by the ethics committee of Isfahan University of Medical Sciences (IR.MUI.NUREMA.REC.1401.112), and taking the necessary permissions, the researchers will select the participants by referring to the research environment. After providing complete explanations regarding the purpose of the study and the method of research, the eligible participants are assigned an appointment in a private and comfortable environment. All participants will ensure that their participation is voluntary, and they are free to discontinue their cooperation whenever they want. After obtaining verbal and written consent, the interviews will be recorded by an MP4 device. In cases where voice recording is not permitted, the interviews will be done by taking notes.

In a face-to-face meeting, data will gather through individual, in-depth, and semi-structured interviews along with taking notes in the field**.** In this method, the first several interviews will be conducted to get familiar with possible and unpredicted issues, as well as guide for subsequent interviews.

The interview guiding questions will consist of open-ended questions based on the main constructs of Bandura’s self-efficacy theory that researcher wants covered in the interview. Directed questions guide how to conduct the interview. In this study, Interviews begin with the guiding questions “How is the experience of motherhood? How much do you manage to take care of yourself and baby?” and “What problems do you have about taking care of yourself, and your baby? What is difficult for you to do? How do you cope with your situation?” and “How do your husband and family take care of you? How much do they give you the feeling that you can handle your situation?”. The directed question for assessing “social modeling” construct will be asked in the form of “can seeing the success of teen mothers like you motivate and stimulate you to do these things too?” or “How do teen mothers take care of their children? Do you think you are like them or more successful than them?”.

Moreover, the following question “Has anyone told you that you can’t do something? or will they tell you that you are more successful in a certain field? What do you do very well in taking care of your child that others tell you?” will be asked for evaluating the successful personal experiences, and social persuasion constructs.

And the questions which asked from health care provider and maternal health policy makers include: “From your point of view, what factors can affect a teen mother’s belief in her ability to manage and adapt to the parental role? Is there a difference between teenage and adult mothers in this regard?” or “what is your challenge in the care of teen mothers? which kind of problems will the care givers be encountered in the care of teen mothers and what are your suggestion?” and “What can be done in order for teen mothers to be able to cope, and manage their needs, and condition? What interventions are more appropriate? Why?”.

What solutions can you offer in order for teen mothers to be able to cope with their work?

The length of the interview will be adjusted according to the willingness of the participant to answer the questions. If needed, a supplementary interview will also be scheduled. After the end of each session, the audio file of the interview will be transcribed immediately, and data analysis will be performed simultaneously with data collection. Data collection will continue until the data saturation, that is when no new code or data is extracted.

### Data management and analysis

Data analysis and interpretation will be deductively done using directed content analysis introduced by Hsieh & Shannon [31]. The existing previous theory or conceptual framework with principal concepts or constructs can provide predictions about the relationships between variables, therefore will assist with the organization and coding of data. In this approach, the previous theory can be validated, and probably expanded and strengthened. The bandura self-efficacy theory will be used to assist with organization and coding of data.

The three main stages, including preparation, organization, and reporting will be applied for directed content analysis. The first phase will begin with carefully reading the whole text, and selecting the unit of meanings. Moreover, the researcher will try to obtain a sense from the whole data and understand “what’s going on”. The organization stage is the development of an unconstrained classification matrix and data coding based on the categorize of model. In this step, similar meaning units will be placed in the constructs of Bandura’s self-efficacy model (successful experiences of the individual, psychological responses, social modeling, and social persuasion). Any section of the text that cannot be categorized by the initial coding scheme will be assigned a new code. In the reporting phase, the researcher will create a link between the results and describe the analysis process in detail. The Max-QDA version 10 software will be used to facilitate, organization and analysis of the qualitative data.

### Rigor and trustworthiness

To assure the rigor, and trustworthiness of the findings, four criteria of Lincoln and Guba, including credibility, dependability, confirmability, and transferability, will apply as the scientific accuracy criteria [32, 33]. In order for credibility, different methods including the member-checking technique (review by the participants), the maximum variation in the participant' selection, in-depth interviews at different times and locations, constant engagement with the research subject, and integrating various data-gathering methods (e.g., individual interviews, focus group discussions, daily notes, and field notes) will be used. The dependability of the findings will be ensured through accurate documentation and recording research details. To guarantee the data confirmability, the external observer’s method will be used to control the accuracy of the coding and theme extraction process and find any contradictory cases. To enhance the transferability, the results will be offered to some women with similar characteristics to the participants, who were not present in the research to judge, and compare the similarity of the findings with their own experiences.

### Second phase: scoping review

In this phase, the postpartum care service packages, programs, guidelines, and protocols, as well as recommendation, strategies, and interventions for postpartum self-efficacy of adolescent mother will be extracted using a scoping review methodology. Using this method enables combining a wide range of studies and summarizing information from a variety of sources and evidence to gain a comprehensive understanding of a given field of study, as well as, inform future research. The 5-step framework developed by the Joanna Briggs Institute will be used for this scoping review [34, 35].

### First step: identify the research question

In the first step, the research question will be identified. The primary questions are “What is the current body of scientific literature regarding the various strategies for promoting adolescence mother self-efficacy”, and “what is the main challenges, and need of adolescence mother in postpartum period”.

### Step 2: identify relevant studies

In the next step, the relevant studies will be identified. For this stage, the original articles published in English and Persian electronic databases including PubMed, Scopus, Embase, Web of Science, Cochrane Database of Systematic Reviews (CDSR), Sid, IRANDOC, and Mag-Iran will be systematically searched for the period up to 30 January 2023 using the following search strategies in accordance with the Mesh browser keywords and free-text words: (((Adolescent[mh] OR Adolescents[tiab] OR Adolescence[tiab] OR Teens[tiab] OR Teen[tiab] OR Teenagers[tiab] OR Teenager[tiab] OR Youth[tiab] OR Youths[tiab] OR Adolescents, Female[tiab] OR Adolescent, Female[tiab] OR Female Adolescent[tiab] OR Female Adolescents[tiab] OR Minor[tiab]) AND (Self-efficacy[mh] OR confidence[tiab] OR self-concept[mh] OR competence[tiab] OR self-confidence[tiab] OR self-perception[tiab] OR confidence[tiab])) AND (Program[mh] OR education program[tiab] OR guideline[tiab] OR service package[tiab] OR protocol[tiab] OR Intervention[tiab] OR health education[tiab] OR client education[tiab] OR women education[tiab] OR Antenatal education[tiab] OR perinatal education[tiab] OR Clinical Trial[mh] OR Evaluation Study[mh] OR Controlled Clinical Trial[tiab] OR Randomized Controlled Trial + [tiab] OR Adaptive Clinical Trial[tiab] OR control*[tiab] OR random*[tiab] OR trial*[tiab] OR effectiveness[tiab] OR efficacy[tiab] OR compar*[tiab] OR clinical*[tiab] OR experiment*[tiab] OR impact evaluation[tiab] OR impact study[tiab] OR impact assessment[tiab] OR outcome evaluation[tiab] OR out-come study[tiab] OR outcome assessment[tiab])) AND (Postpartum Period[mh] OR Postpartum[tiab] OR Postpartum Women[tiab] OR puerperium[tiab] OR Child[tiab] OR newborn[tiab] OR infant[mh] OR infancy[tiab] OR parent[tiab] OR mother[tiab] OR pregnancy[mh] OR post-natal[tiab] OR neonate[tiab] OR neonatal[tiab]). In addition, hand-searching in gray literature databases (e.g., Google Scholar, Global Index Medicus (GIM), Open Grey SIGLE, World Cat, NTIS, UW Libraries Search) and screening reference lists of eligible articles will be used to find more relevant records. All type of articles including quantitative, qualitative, and mixed methods studies will be assessed. The Endnote software ver.X9 will be used for omitting duplicate studies in different databases.

### Step 3: study selection

After checking the titles and abstracts of all articles, the full text of the studies will be evaluated to identify the articles that met the inclusion criteria. All observational, and interventional studies, and programs will be assessed based on the needs, recommendation and strategies for self-efficacy promotion of teen mother during postpartum period.

### Step 4: charting the data

After finding the full texts of relevant articles, data of each article including the author's name, publication date, type of studies, setting, the sample size, intervention development, the time of the interventions, measurement tool, the needs, recommendation and strategies for promoting self-efficacy of teen mother, and main findings will be extracted using a structured form. Using the results of a scoping review, the needs, solutions and strategies for improving the self-efficacy of teen mothers will be extracted.

### Step 5: collating and summarizing of result

In this step, the quantitative and qualitative information of each relevant article will be analyzed separately by two reviewers.

#### The third phase: designing a postpartum self-efficacy promotion program

In this stage of the research, after extracting the self-efficacy needs of teen mothers through qualitative study and scoping review, the decision matrix will be used to prioritize the strategies. In the first round of Delphi, a number of specialists (including obstetricians, reproductive health experts, child psychologist, maternal health policymakers, youth health policymakers) will assign to each proposed strategy a score of 1 to 9 based on four criteria including cost, time spent, place of implementation and feasibility. A score of 9 corresponds to the option “It is expected benefits are greater than its harms” and a score of 1 is related to the option “It is expected harms are much greater than its benefits”. The average score for each question will be obtained, if the score is between 1 and 3, it will be considered as an inappropriate option and will be removed from the program. Scores from 7 to 9 will be entered into the program as suitable options, and scores from 3 to 7 will be entered into the second round of Delphi to achieve general consensus.

In the second round of Delphi, experts will be invited again to attend the face to face meeting to discuss the prioritization of strategies, the components of the program, its structure and application, the team members involved in it and other points of disagreement in the first Delphi phase. In the end, based on the panel members’ views, Comments and suggestions will be collected and applied to the design of the intervention program, and subsequently finalized and implemented in the quantitative phase.

## The fourth phase: quantative phase

### Type of quantitative study

The quantitative phase of the research will be carried out using a two-group semi-experimental study.

### Research population

The targeted population is all adolescent mothers referred to health care centers affiliated to Isfahan University of Medical Sciences.

### Research sample

The study sample will be formed from a group of teen mothers who will be selected by convenience sampling method and have all inclusion criteria for the study.

### Research environment

This study will be carried out in selected comprehensive health care centers, obstetrics and gynecology offices, perinatal clinics of hospitals and all places where teen mothers are accessible. The reason for choosing such kind of environment is easy access to teen mothers and includes the majority of cultural and social classes. Considering that the present research is a mixed method study, the type and time of the intervention will be determined based on the findings of the previous stage, but our prediction is that the intervention will start from pregnancy.

### Sampling method, and sample size

The sample size will be calculated using a pilot study. The sampling will be conducted by convenience and non-probabilistic sampling methods. The researcher by referring to the research environment on consecutive days will choose the number of teen mothers who have inclusion criteria in the easy method. Thereafter, participants will assign to the intervention and control groups based on ALLOCATION and RANDOM software. The sample size will be calculated based on pilot study.

### Inclusion criteria

Pregnant women aged between 15 and 19 years, pregnancy age over 36 weeks, low-risk singleton pregnancy, Iranian citizenship, consent to participate in the study, not having history of any diagnosed mental or chronic diseases undergoing drug treatments, and ability to read and write.

### Exclusion criteria

Unwillingness to continue cooperation during the study, occurrence of high-risk obstetric conditions (e.g., pre-eclampsia, premature birth, hysterectomy, etc.), failure to receive 50% of the intervention for any reason.

### Study variables

In this study, the designed interventions are considered as an independent variable, and postpartum self-efficacy is considered to be the dependent variable. Variables affecting self-efficacy will be determined using different constructs of Bandura’s self-efficacy theory (e.g., including successful experiences of the individual, social modeling, social persuasion, and psychological responses), and based on the results of a qualitative study and scoping review, different areas of self-efficacy (e.g., self-efficacy in self-care, infant care, breastfeeding, etc.) will be determined.

### Data collection method

Researcher-made questionnaires evaluating postpartum self-efficacy in adolescent mother will be applied in the quantitative phase of this trial. After qualitative interview and searching in existing questionnaire, the validity and reliability of this questionnaire will be determined. In order to assess the content and face validity, the scale will be given to 15 faculty members of the midwifery, gynecology, and psychology department, and their expert’ opinions will be qualitatively received and the suggested changes will be applied in the questionnaire. In order to check face validity, the questionnaire will be provided to a number of women with the characteristics of the research units. The reliability of the tool will be done by test–retest method and the internal correlation will be evaluated with Cronbach’s alpha test.

### The implementation method

Based on the opinion of expert panel, a part of the strategies and goals will be determined in the form of an intervention program. After approving the protocol of the study with Ethics Committee, and obtaining the necessary coordination between the authorities of healthcare centers and hospitals, the researcher will implement the designed interventional program. After explaining the aim of the study (by Samaneh Youseflu), verbal, and informed consent will be given from the eligible women, and if they agree, they will enter the study and be randomly divided into two groups. For sampling, eligible women will be entered into the study using the convenience sampling method. Then, for randomized assignment, the women will randomly assign either to the trial or control groups based on the table of random numbers that will be generated by Random Allocation Software 2.0. To prevent selection bias, allocation concealment to the trial or control group will be done by a researcher who does not participate in the data collection (Shahnaz Kohan).

At first, in the 36th week of pregnancy, a pre-test will be taken from both groups and the average self-efficacy scores will be compared between the two groups. The intervention group received the self-efficacy promotion program and the control group did not receive any intervention. After delivery, their self-efficacy score will be checked between two groups. The type of intervention (e.g., role playing, peer education, home visit, virtual training, and etc.), the time of the program implementation (pregnancy, after childbirth, etc.), the people involved in the program will be determined based on the opinion of the expert panel.

### Data analysis

The data analysis will be done using SPSS software ver. 16 by descriptive, and inferential statistics including paired t-test, repeated measure ANOVA, Chi-squared test, Fishers exact test, ANOVA, Wilcoxon test, and Mann–Whitney test.

## Discussion

Adolescent pregnancy can encounter many challenges for the teen mother, family, and health system. Similar to the increase in the global fertility rate in adolescent pregnancy, it is expected that due to human population planning policies in Iran and the provision of childbearing incentives, in the not too distant future, Iran will experience an increasing growth of adolescent pregnancy rates. This matter highlights the urge to develop comprehensive reproductive health care program in order to care for themselves, their infants, and family. Therefore, the current mixed methods study will be conducted as a sequential exploratory approach to provide an interventional program sensitive to the culture of the Iranian society for adolescent mothers in postpartum care. The combination of qualitative and quantitative research methods will result in a more comprehensive view of the self-efficacy needs of teen mothers, and developing programs in postpartum care.

For a teen mother, the simultaneity of two transition periods, i.e. transition to adulthood and transition to the post-partum period, disrupts the acceptance of new roles. The transition from one stage to another is associated with instabilities and requires the acquisition of skills and abilities to adapt to new roles. Teen mother should be able to balance between multiple roles and mood changes. Therefore, a healthy transition requires mental well-being, mastery of roles and good and logical relationships. On the other hand, having a healthy transitional stage depends a lot on the amount of information, the type of attitude and acquired behaviors. Lack of knowledge, information and life skills in managing this situation may threaten the physical and mental health of the mother, family and children and reduce the level of role adaptation. Previous researches show that in adolescent mother, the perceived stress following pregnancy and childbirth is higher than adults; moreover, the perceived stress after childbirth is much higher than pregnancy. Therefore, it seems critical to develop fundamental culturally sensitive community-based interventions in accordance with the biological, cognitive, psycho-social structure of teen mother in order to increase their self-efficacy.

Despite the influence of the adolescent health in many health indicators, investing, and planning in various aspects of their health is often overlooked. Shortage of knowledge of maternal health care providers in the caring of teen mothers along with lack of comprehensive guidelines are the main reasons for neglecting them. By implementing this program, teen mothers can access postpartum services that are appropriate for their age characteristics. Moreover, in this program, in addition to improve the health of mothers, they will also improve their self-efficacy. In the meantime, the conduct of this program is considered as a logical and efficient solution for improving postpartum outcomes, and reduces their medical and treatment costs.

Developing an interventional program that includes mother’s experiences, evidence based practice principles, and health staff opinions in a distinct cultural and social context can supply new direction to lead manager, policymakers, and other health care provider to improve the maternal and child health outcome, as well as quality of postpartum care for teen mothers with considering their age characteristics.

## Data Availability

The data sets will be used and analyzed during the current study are available from the corresponding author upon reasonable request.
